# CD4^+^ T Cell-Dependent Macrophage Activation Modulates Sustained PS Exposure on Intracellular Amastigotes of *Leishmania amazonensis*

**DOI:** 10.3389/fcimb.2019.00105

**Published:** 2019-04-12

**Authors:** Joao Luiz Mendes Wanderley, Poliana Deolindo, Eric Carlsen, Arieli Bernardo Portugal, Renato Augusto DaMatta, Marcello Andre Barcinski, Lynn Soong

**Affiliations:** ^1^Laboratório de Imunoparasitologia, Unidade de Pesquisa Integrada em Produtos Bioativos e Biociências, Universidade Federal do Rio de Janeiro, Macaé, Brazil; ^2^Laboratório de Biologia Molecular de Parasitas e Vetores, Fundação Oswaldo Cruz, Rio de Janeiro, Brazil; ^3^Department of Pathology, University of Pittsburgh Medical Center, Pittsburgh, PA, United States; ^4^Laboratório de Biologia Celular e Tecidual, Universidade Estadual do Norte Fluminense, Campos dos Goytacazes, Brazil; ^5^Instituto de Biofísica Carlos Chagas Filho, Universidade Federal do Rio de Janeiro, Rio de Janeiro, Brazil; ^6^Department of Microbiology and Immunology, Center for Tropical Diseases, Institute for Human Infections and Immunity, University of Texas Medical Branch, Galveston, TX, United States

**Keywords:** phosphatidylserine, amastigote, T cell, parasitophorous vacuole, macrophage, immune evasion

## Abstract

*Leishmania amazonensis* amastigotes can make use of surface-exposed phosphatidylserine (PS) molecules to promote infection and non-classical activation of macrophages (MΦ), leading to uncontrolled intracellular proliferation of the parasites. This mechanism was quoted as apoptotic mimicry. Moreover, the amount of PS molecules exposed on the surface of amastigotes correlates with the susceptibility of the host. In this study, we tested whether host cellular responses influence PS expression on intracellular amastigotes. We found that the level of PS exposure on intracellular amastigotes was modulated by CD4^+^ T cell and MΦ activation status *in vitro* and *in vivo*. *L. amazonensis* infection generated a Th1/Th2-mixed cytokine profile, providing the optimal MΦ stimulation that favored PS exposure on intracellular amastigotes. Maintenance of PS exposed on the parasite was dependent on low, but sustained, levels of nitric oxide and polyamine production. Amastigotes obtained from lymphopenic nude mice did not expose PS on their surface, and adoptive transfer of CD4^+^ T cells reversed this phenotype. In addition, histopathological analysis of mice treated with anti-PS antibodies showed increased inflammation and similarities to nude mouse lesions. Collectively, our data confirm the role of pathogenic CD4^+^ T cells for disease progression and point to PS as a critical parasite strategy to subvert host immune responses.

## Introduction

*Leishmania amazonensis* (*L. amazonensis*) is the causative agent of cutaneous Leishmaniasis in South America. This species is associated with most cases of diffuse/disseminated cutaneous Leishmaniasis (DCL), a very severe clinical manifestation (Leon et al., 1990). Experimentally, most inbred mouse strains develop progressive cutaneous lesions, although the disease severity varies among mice of different genetic backgrounds (Terabe et al., 2004). Both DCL patients and experimentally infected mice show deficient cellular immune responses to the pathogen, as judged by delayed-type hypersensitivity (DTH) responses or cytokine/chemokine profiles (Ji et al., 2003; Silveira et al., 2005). In fact, when compared to the classical *L. major* infection models, *L. amazonensis*-infected mice failed to elicit a polarized Th response because activated CD4^+^ T cells produced a mix of Th1/Th2/Th17 and modulatory cytokines (Ji et al., 2003; Ramer et al., 2006; Vargas-Inchaustegui et al., 2009). This phenotype is consistent with the poor activation presented by infected dendritic cells, which is not sufficient to turn these cells into efficient, functional antigen-presenting cells (Xin et al., 2008; Wanderley et al., 2013). Consequently, macrophages (MΦs), the preferential host cells for parasite growth, are not efficiently activated and not capable of controlling the infection. Competent MΦ activation is necessary for disease control, since those cells are the main effector cells for parasite killing, usually dependent on the expression of nitric oxide synthase (iNOS) (Xie et al., 1993) or the production of reactive oxygen species (Carneiro et al., 2018). On the other hand, alternative or non-classical MΦ activation leads to an increased activation of arginase I, an enzyme responsible for the first step of polyamine synthesis, which is mandatory for parasite growth (Franca-Costa et al., 2015) and restrains NO production by competing for the same substrate, L-arginine (Wanasen and Soong, 2008). Those intracellular pathways control the fate of the intracellular parasite.

Apoptotic cells are known to display several distinctive molecular patterns, which are recognized by phagocytic cells for efficient internalization (Poon et al., 2014). In addition, phagocytes stimulated by apoptotic cell recognition are prompted to produce modulatory cytokines such as TGF-β and IL-10 (Fadok et al., 1998). Phosphatidylserine (PS) is a structural phospholipid that is actively maintained in the cytoplasmic leaflet of the plasma membrane but is translocated to the surface at the early stages of apoptotic death (Fadok et al., 1992). Recognition of PS exposed at the surface of apoptotic cells is sufficient to induce apoptotic cell clearance and non-classical activation of phagocytic cells (Hoffmann et al., 2001). We had previously shown that the amastigote forms of *L. amazonensis*, when purified from mice lesions, exposed PS at their surface without additional signs of apoptotic death. Since PS-exposing amastigotes are fully viable and highly competent in infecting and maintaining a productive disease in mice, we termed this phenotype apoptotic mimicry (de Freitas Balanco et al., 2001). As in the case of apoptotic cell/phagocyte interactions, the host cell is induced to produce immunosuppressive cytokines, which, in turn, signal for MΦ non-classical activation and consequent parasite growth (de Freitas Balanco et al., 2001; Wanderley et al., 2006). PS exposure on *L. amazonensis* amastigotes correlates with the severity of the disease, since amastigotes purified from BALB/c mice, which are highly susceptible to the infection, exhibit a higher density of PS moieties than do those from parasites purified from semi-resistant C57BL/6 mice (Wanderley et al., 2006). In addition, *in vivo* treatment of infected mice with anti-PS monoclonal antibodies delays disease progression and up-regulates the efficiency of dendritic cells to present antigen and activate parasite-specific T cells (Wanderley et al., 2013). PS exposure on pathogens operates in several different models of infection, such as those using *Trypanosoma cruzi* (Damatta et al., 2007), *Toxoplasma gondii* (Seabra et al., 2004), enveloped and non-enveloped viruses in which they confirm PS as a strategy to silently invade host cells (Seabra et al., 2004; Damatta et al., 2007; Mercer and Helenius, 2008; Feng et al., 2013). Additionally, by inducing transient PS exposure on the surface of host cells, viral infections can spread signals derived from PS recognition, such as TGF-β and IL-10 production by neighbor phagocytes, to avoid full activation of the immune system (Soares et al., 2008).

In this study, we tested whether PS exposure is an adaptive response of *L. amazonensis* amastigotes to the hostile environment of the parasitophorous vacuole generated by MΦ immune activation. We observed that intracellular amastigotes infecting activated MΦs are able to increase PS exposure. This is dependent on iNOS and arginase I concomitant expression. We confirmed our findings by demonstrating that PS exposure on amastigotes purified from lesions of T cell-deficient nude mice was nearly absent, but the adoptive transfer of primed CD4^+^ T cells recovered this phenotype. We also demonstrated that lesions of anti-PS antibody-treated infected mice were similar to lesions of immunodeficient mice. Our data lead us to conclude that PS exposed by intracellular amastigotes of *L. amazonensis* is a phenotype acquired as a response to host immune activation, and thus an important adaptive strategy employed by those intracellular parasites.

## Materials and Methods

### Mice and Parasites

Female nude BALB/c mice (C.Cg/AnNTac-*Foxn1*^*nu*^ NE9), C57BL/6 mice deficient in iNOS (C57BL/6NTac-Nos2^tm^1N12), and their corresponding wide-type (WT) controls were purchased from Taconic Farms (Germantown, NY) or Harlan Sprague Dawley (Indianapolis, IN), respectively. All mice were maintained under specific pathogen-free conditions and used at 6–8 weeks of age, according to the protocols approved by the Animal Care and Use Committee of the University of Texas Medical Branch (#9803016A). Promastigotes of *L. amazonensis* (LV78) were cultured at 23°C in Schneider's *Drosophila* medium (Invitrogen, Carlsbad, CA), pH 7.0, supplemented with 20% FBS (Sigma, St. Louis, MO) and 50 μg/ml of gentamicin. Axenic amastigotes of *L. amazonensis* (LV78) were cultured at 33°C in complete Grace's insect cell culture medium (Invitrogen), pH 5.0, supplemented with 20% FBS. Parasite infectivity was maintained by *in vivo* passages in BALB/c mice, and cultures of <6 passages were used for infection.

### Reagents

Otherwise stated, all recombinant cytokines were purchased from Peprotech (Rocky Hill, NJ, USA). Superoxide scavenger MnTBAP (Mn^3^ tetrakis (4-benzoic acid) porphyrin chloride) was purchased from Enzo Life Sciences (Farmingdale, NY, USA), iNOS inhibitor L-NIL- [L-N^6^-(1iminoethyl) lysine], and (ODC) decarboxylase inhibitor DFMO (DL-α-Difluoromethylornithine, Hydrochloride) were purchased from Calbiochem (Darmstadt, Germany).

### Amastigote Purification

Infected tissues or infected MΦs were finely minced and homogenized with a tissue grinder (Thomas Scientific, NJ). The cell suspension was centrifuged at 50 g for 10 min at 4°C. The supernatant was carefully collected, and further centrifuged and washed for 3 more times at 1,450 g for 17 min at 4°C. After 2 h incubation under rotation at 34°C to liberate endocytic membranes (Saraiva et al., 1983), amastigotes were further centrifuged and incubated for 16 h at 34°C to complete release of endocytic membranes and to test for bacterial contamination. After this time, they were centrifuged and washed 3 times before use. Prior to amastigote purification from *in vitro* infected cells, MΦs were thoroughly washed with HBSS.

### Generation of Bone Marrow-Derived Macrophages (BMMΦs)

BMMΦs were generated from mice by cultivating fresh bone marrow cells in complete IMDM (Invitrogen) containing 10% FBS, supplemented with 20 ng/ml of recombinant M-CSF (eBioscience, San Diego, CA). To generate BMMΦs, we replaced the medium at 5–6 days of culture and harvested adhered cells after 10–12 days. To recover adhered MΦs, we washed the petri dishes twice with warm PBS (Invitrogen) and incubated the cells with 5 ml of cell dissociation solution (CellGro, Manassas, VA) for 20 min at room temperature. We detached the cells by pipetting up and down and washed the cell pellet twice with complete medium prior to use.

### Flow Cytometry

Parasites were quantified and 10^6^ amastigotes were washed and suspended in annexin V binding buffer, which contains 10 mM HEPES, 150 mM NaCl, and 2.5 mM CaCl_2_, at pH 7.2. Cells were incubated at room temperature for 15 min with annexin V-FITC (Molecular Probes, Eugene, OR) at the concentration indicated by the manufacturer and diluted in the binding buffer. All incubation procedures were performed on ice. At the time of acquisition, 0.4 μg/ml of propidium iodide (PI, Sigma) was added to the control and Annexin V-FITC-labeled samples to determine parasite viability. Data were collected in a BD FACSCalibur® (20,000 gated events per sample) and analyzed by Cellquest Pro® (BD Biosciences, San Jose, CA) and FlowJo software (TreeStar, Ashland, OR).

### Generation of Supernatant (SN) From Stimulated Lymph Node Cells

Mice were infected in the footpad with 2 × 10^6^ promastigote forms of *L. amazonensis*. After 5–7 weeks of infection, popliteal lymph nodes (LNs) were harvested, and a single-cell suspension was obtained. Total LN cells from infected or naïve mice were plated in U-bottomed, 96-well plates, 4 × 10^5^ cells per well, in the presence of 40 μg/ml of soluble *Leishmania*l antigens (SLA). After 4 days of culture, supernatants from stimulated LN cells from naïve mice or infected mice were pooled, filtered, and stored in aliquots at −70°C. To generate SLA, promastigote forms were submitted to 5 cycles of freeze-and-thaw and centrifugation to dispose of insoluble materials.

### Adoptive Cell Transfer

BALB/c WT or nude mice were infected in the footpad with 1–2 × 10^6^ promastigote forms of *L. amazonensis*. After 6–8 weeks of infection, popliteal LNs from WT mice were harvested and a single-cell suspension was obtained. CD4^+^ or CD8^+^ T cells were purified by selected cell isolation kits (Miltenyi Biotech, Alburn, CA), following the manufacturer's instructions. Infected BALB/c nude mice were i.v. injected with 1–3 × 10^6^ purified CD4^+^ or CD8^+^ T cells. At 2–3 weeks post-transfer, mice were euthanized to obtain popliteal LNs and lesion-derived amastigotes to evaluate T cell activation and PS exposure on amastigotes, respectively.

### Macrophage Infection

Thioglycolate-elicited peritoneal MΦs or BMMΦs were placed on 24-well plates and allowed to attach overnight. Cells were incubated with axenic amastigotes or promastigotes in a 3:1 ratio. After 4 h at 33°C, free parasites were removed by washing and, if necessary, cells were activated and/or treated with SNs, cytokines or drugs. Cultures proceeded for an additional 24-h period. In some cases, MΦs were attached on 13-mm^2^ glass coverslips (Fisher Scientific, Pittsburgh, PA) and, after infection and activation/treatment, were stained with Giemsa (Sigma) to evaluate host cell morphology and infection efficiency.

### Polymerase Chain Reaction (PCR)

Total RNA was extracted from 1 × 10^6^ MΦs 24 h post-infection and/or activation by using the RNeasy system (QIAGEN, Valencia, CA). Immediately cDNA was generated by using up to 5 μg of total RNA and the Superscript III Synthesis System (Invitrogen) and following the manufacturer's instructions. Amplifications of specific cDNAs were performed by using the GoTaq® Green Master Mix system (Promega, San Luis Obispo, CA). Briefly, Arginase I cDNA was subsequently amplified by use of the following cDNA primers: sense, 5′-AGACATCGTGTACATTG-3′ and antisense, 5′-GAGTTCCGAAGCAAGCCAAG-3′. Amplification occurred over 30 cycles, with the first cycle for primary denaturing at 95°C for 2 min; the next 28 cycles each comprising three steps for denaturing (94°C, 35 s), primer annealing (59°C for 45 s) and primer extension (72°C, 45 s); and a final cycle of denaturing (95°C, 30 s), annealing (69°C, 30 s), and extension (72°C, 5 min). To amplify inducible nitric oxide synthase (iNOS) and β-actin cDNA, we used the following cDNA primers: iNOS sense, 5′-GTTTCTGGCAGCAGCGGCTC-3′; antisense, 5′-GCTCCTCGCTCAAGTTCAGC-3′. β-actin sense, 5′-CGTGGGCCGCCCTAGGCACCAGGG-3′; antisense, 5′-GGGAGGAAGAGGATGCCGCAGTGG-3′. Amplification occurred over 36 cycles, by using the following approaches:the first cycle for primary denaturing at 95°C for 2 min; 34 cycles each comprising three steps for denaturing (95°C, 30 s), annealing (69°C, 30 s), extension (72°C, 20 s); and a final cycle of denaturing (95°C, 30 s), annealing (69°C, 30 s), and extension (72°C, 5 min). All reactions were performed by using a GeneAmp PCR System 2700 (Applied Biosystems, Foster City, CA), and the PCR products were separated by electrophoresis on 1.2% agarose gels. Real-time RT-PCR assays were performed with TaqMan Universal PCR Master Mix (Applied Biosystems, Foster City, CA, USA), using the following primer-probe sets purchased from Applied Biosystems: *inos* (Mm00440502_m1), *arginase I* (Mm00475988_m1), and β*-actin* endogenous control. The reactions were performed using Bio-Rad CFX96 Real-Time PCR detection system. Data were normalized to the expression of β*-actin*.

### Cytokine Production

Cytokine production was measured in the supernatant of LN cell or MΦ cultures by using the Bio-Plex Pro-Mouse Cytokine 23-plex Assay from Bio-Rad (Hercules, CA) and following the manufacturer's instructions. Total and biologically reactive TGF-β_1_ were measured by using the ELISA Ready-SET-Go system (eBioscience), and data for biological reactive TGF-β_1_ are presented, The level of nitric oxide was measured by using a Griess assay (Caymann Chemical, Ann Arbor, MI).

### Parasite Quantification by Real-Time PCR

Parasite loads were quantified by measuring the gene of *L. amazonensis* cysteine proteinase isoform 1 (*Llacys1*), which is a single-copy gene per haploid genome and expressed in both developmental stages (Lasakosvitsch et al., 2003). Infected MΦs were collected for DNA extraction with a DNeasy kit (Qiagen, Valencia, CA). DNA (10 ng) was used for parasite detection by the UTMB Real-time PCR Core Facility (all reagents were purchased from Applied Biosystems, Foster City, CA). Each sample was run in duplicate and normalized by the amount of total DNA extracted. The number of parasites per sample was calculated based on a standard curve, as described in our previous studies (Xin et al., 2010).

### Histopathological Analysis

Mice were infected i.d. in the right ear with 10^6^ promastigotes. After 2 weeks of infection and every 3 days thereafter they were given i.p. injections of 100 μg of PGN635, a second-generation fully humanized anti-PS monoclonal antibody (Zhou et al., 2014). Other groups of mice received PBS or the isotype control C44 antibody that binds to colchicine (Edmond Rouan et al., 1989). Mice were treated for 6 weeks and the infected ears were collected, fixed in 4% paraformaldehyde, dehydrated, embedded in paraffin, and mounted slides were stained with hematoxylin and eosin.

### Parasitophorous Vacuole Morphometric Evaluation

The sizes parasitophorous vacuoles from lesions of mice treated with anti-PS, isotype antibodies or PBS were observed under an Axioplan (Zeiss) microscopy and images were captured using a MRc5 AxioCam digital camera and processed with the software ImageJ version 1.47t (Wayne Rasband–NIH). Values are shown as the area in μm^2^ for at least 200 PVs in each tested sample.

### Western Blot

BMMΦs (1 × 10^6^) were infected with axenic amastigotes at a 3:1 parasite-to-cell ratio. At 24 h post-infection and/or indicated treatments, cells were harvested, washed, and suspended in 10 μl of PBS and 10 μl of 2X lysis buffer (2% Triton X-100, 100 mM Tris-Cl, 600 mM NaCl, 10 mM EDTA, 2 mM PMSF, 250 mM sucrose) that contained an inhibitor cocktail (Roche, Indianapolis, IN). Protein concentrations were determined by using the BCA protein assay kit (Pierce Biotechnology). Equal amounts of proteins were loaded onto 10% SDS-polyacrylamide gels, and then transferred to polyvinylidene difluoride membranes (BioRad Laboratories, Hercules, CA). Rabbit anti-mouse iNOS and arginase I antibodies were purchased from Santa Cruz Biotechnology (Santa Cruz, CA). Mouse anti-actin mAb (Sigma) was obtained from Dr. Jiaren Sun (Department of Microbiology and Immunology, UTMB, TX). Membranes were incubated with primary Abs (diluted 1:200 in TBS buffer containing 5% non-fat milk and 0.05% Tween-20) at 4°C overnight, washed, and incubated with an HRP-conjugated secondary Ab (1:2000) for 1 h. Blots were developed with the enzyme chemiluminescence kit ECL (Amersham Biosciences, Piscataway, NJ).

### Statistical Analysis

One- or two-way ANOVA was used for multiple group comparisons (GraphPad Software v5.0, San Diego, CA). Statistically significant values are referred to as follows: ^*^, *p* < 0.05; ^**^, *p* < 0.01; ^***^, *p* < 0.001.

## Results

### Increase of PS Exposure on Intracellular Amastigotes Depends on Macrophage Interactions With Lymph Node Cells

**Figure 1:**
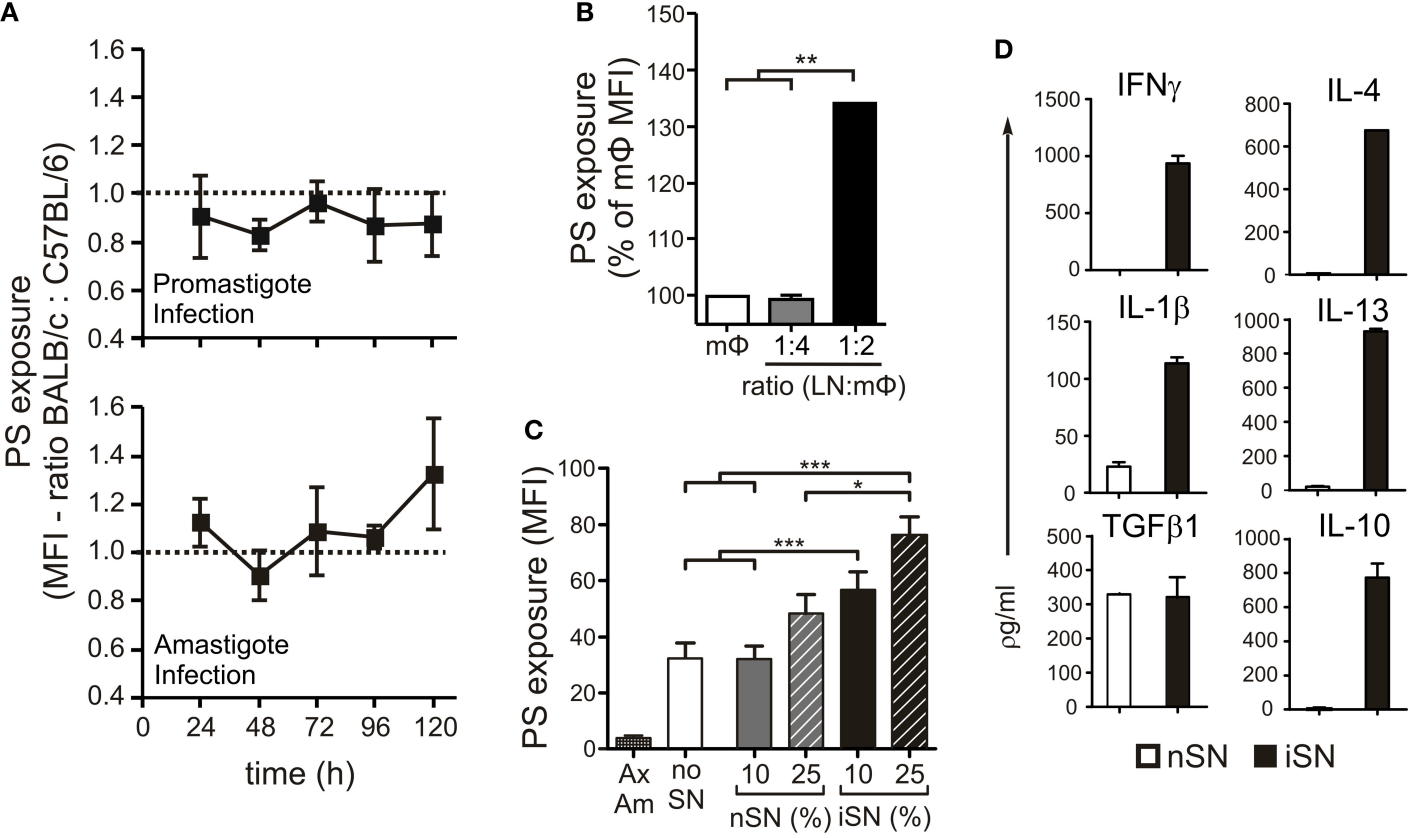
PS exposure on the surface of intracellular amastigotes is induced through MΦ activation by lymph node cells. **(A)** BALB/c- or C57BL/6-derived peritoneal MΦs were infected with stationary-phase promastigotes (upper panel) or amastigotes (lower panel). Every 24 h post-infection, intracellular parasites were purified, and PS exposure was analyzed by flow cytometry. Results are shown as the ratio between the MFIs of annexin V staining of amastigotes derived from BALB/c and C57BL/6 MΦs. BALB/c-derived BMMΦs were infected with amastigotes in the absence or presence of **(B)** LN cells from infected BALB/c mice (at indicated LN cell-to-MΦ ratios), or **(C)** supernatants of *in vitro*-stimulated LN cells from naïve BALB/c mice (nSN) or infected mice (iSN). After 24 h of treatment, amastigotes were purified for PS exposure analysis by flow cytometry. Results are pooled from 3 to 5 independent experiments. **(D)** Cytokine levels in supernatants of *in vitro*-stimulated LN cells from naïve BALB/c mice (nSN) or infected mice (iSN) were measured by bioplex assays. Graph represents data from 3 independent batches of SNs, produced from LNs of 2 mice per batch. *p < 0.05, **p < 0.01, ***p < 0.001.

We previously showed that lesion-derived amastigotes purified from BALB/c mice expose higher amounts of PS than do those parasites derived from C57BL/6 mice (Wanderley et al., 2006), a finding that may indicate that the host can modulate this phenotype of the parasite. To evaluate the role of host macrophages (MΦs) in modulating PS exposure on the parasite, we obtained thioglycollate-elicited peritoneal MΦs from BALB/c and C57BL/6 mice, infected them either with promastigotes or lesion-derived amastigotes and collected intracellular amastigotes every 24 h to evaluate PS exposure by flow cytometry. We found no major differences between parasites derived from BALB/c vs. C57BL/6 MΦs (Figure 1A), regardless of the form of parasite used for infection or the time post-infection. Consequently, we suspected that the interaction between MΦs and other types of immune cells was responsible for the differential levels of PS on lesion-derived parasites *in vivo*. To investigate this possibility, we obtained lymph node (LN) cells from infected BALB/c mice and incubated them with peritoneal MΦs infected with lesion-derived amastigotes. These interactions stimulated an about 30% higher PS exposure on intracellular amastigotes compared to that in parasites obtained from isolated MΦs, in a dose-dependent manner (Figure 1B).

To determine whether cytokine production by LN cells was a requirement to induce PS exposure on intracellular parasites, we generated supernatants from LN cells obtained from naïve (nSN) or infected (iSN) mice and stimulated with SLA for 4 days. We treated MΦs infected with axenic amastigotes with different concentrations of those supernatants (SNs) and evaluate PS exposure on intracellular amastigotes 24 h post-infection and stimulation. As shown in Figure 1C, axenic amastigotes exposed very low amounts of PS, which represented a technical advantage for minimizing background levels of PS exposure and indicated that parasite-host interactions should be necessary to stimulate PS exposure on amastigotes. Indeed, intracellular amastigotes from unstimulated MΦs (“no SN”) increased PS on their surface, 24 h post-infection. In addition, treatment of infected MΦs with iSNs induced PS exposure on intracellular amastigotes. This effect was dependent on the activation of those LN cells, since iSN was significantly more efficient than nSN and a clear positive correlation with the concentration of SN was observed: 10% of iSN added to the culture induced a 50% increase, while 25% of iSN induced about a 70% increase in PS exposure on intracellular amastigotes (Figure 1C).

**Figure 2:**
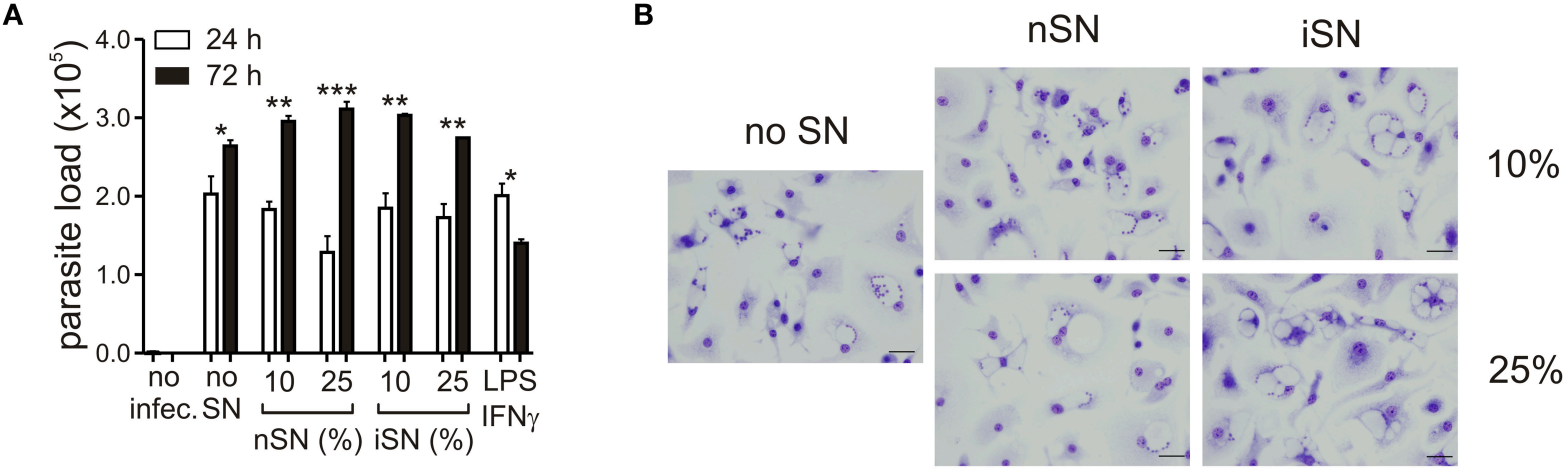
PS exposure on intracellular amastigotes, induced by MΦ stimulation is not due to apoptotic death. **(A)** Parasite loads in infected BALB/c-derived BMMΦs activated with different concentrations of nSN and iSN were measured by real-time PCR. Asterisks indicate comparison between black and white bars. LPS (100 ng/ml) plus IFN-γ (100 ng/ml) was used as positive control. ^*^p < 0.05, ^**^p < 0.01, ^***^p < 0.001. All comparisons were made among the same samples at 24 and 72 h post infection. **(B)** Photomicrography of infected BMMΦs activated with different concentrations of nSN and iSN. Bars indicate 10 μm. Graph represents data from 5 independent experiments.

As expected, the cytokine profile of those SNs corroborated the previous data regarding T cell activation during *L. amazonensis* infection in the mice (Ji et al., 2003). Moderate amounts of Th1, Th2, and modulatory cytokines such as IL-4, IL-13, IFNγ, IL-1β, TGF-β_1_, and IL-10 (Figure 1D), were present, especially in the SN generated from re-stimulated, *in vivo*-primed cells (iSN), which indicates that this response is antigen-specific. One of the hallmarks of the apoptotic mimicry, described to operate during amastigote infection, is the fact that amastigotes exposing PS are perfectly viable and infective and do not display any other sign of apoptotic death (de Freitas Balanco et al., 2001; Wanderley et al., 2006). To evaluate whether MΦ activation leads to PS exposure on intracellular amastigotes due to apoptotic mimicry, we measured parasite loads on infected MΦs at 24 and 72 h post-infection. As shown in Figure 2A, MΦ activation with SNs derived from stimulated LN cells from either infected or naïve mice promoted parasite proliferation 72 h post-infection, as opposed to LPS and IFNγ activation. In addition, the morphological characteristics of *L. amazonensis* infection are maintained, and include, for example, enlarged parasitophorous vacuoles with parasites attached to the vacuole internal membrane (Figure 2B). Our data suggest that MΦ activation through interactions with primed LN cells and their soluble products modulate active PS exposure by intracellular amastigotes in an apoptotic mimicry fashion.

### Balanced Expression of iNOS and Arginase I Controls cytokine-Dependent PS Exposure on Amastigotes and Parasite Survival

**Figure 3:**
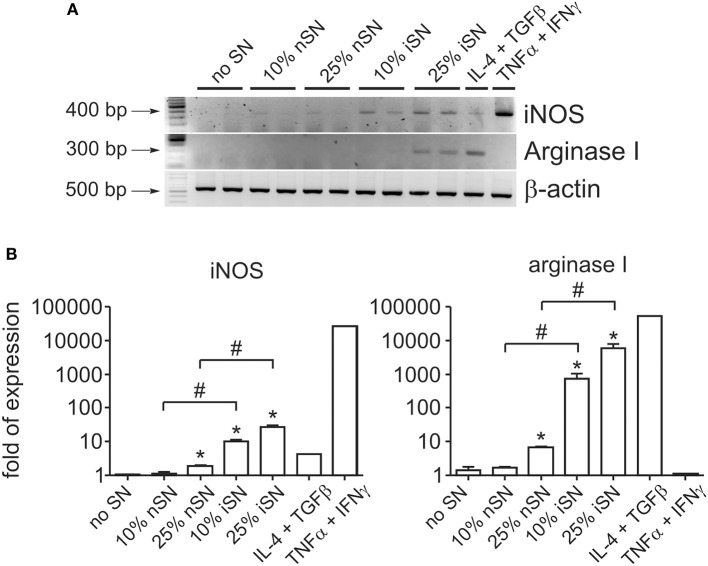
Macrophage stimulation leads to concomitant expression of iNOS and arginase I. **(A)** RT-PCR and **(B)** real-time RT-PCR analyses of iNOS and Arginase I expression by BALB/c-derived BMMΦs at 24 h post-infection and activation with SNs from SLA-stimulated, naïve LN cells (nSN) or re-stimulated, *in vivo*-primed LN cells (iSN). **(A)** Representative photographs of 2–3 experiments. TGF-β (10 ng/ml) plus IL-4 (1 ng/ml) stimulation was used as a positive control for arginase expression and IFN-γ (100 ng/ml) plus TNF-α (10 ng/ml) stimulation for iNOS expression. ^*^p < 0.05 (compared with medium controls), ^#^*p* < 0.05 (between the groups).

It is known that the survival of *Leishmania* parasites inside macrophages (MΦs) is mostly mediated by the balance between iNOS and arginase I activation. These are induced enzymes and therefore their activity is directly related to their cellular expression. Arginase I is the first enzyme in the polyamine synthesis pathway, while iNOS is the enzyme responsible for all steps of NO synthesis. Those intracellular signaling pathways compete with each other, since they depend on the same substrate. In addition, some molecules, produced as secondary metabolites, act as cross-inhibitors (Wanasen and Soong, 2008). Interactions between infected MΦs and other immune cells determine which pathway is activated, since inflammatory cytokines stimulate iNOS expression and decrease mRNA levels of arginase I and vice-versa (Corraliza et al., 1995; Modolell et al., 1995). Since MΦ activation seems to be important for the cytokine-dependent PS exposure by intracellular parasites, we aimed to understand the role of those pathways in this mechanism. We observed that SN from re-stimulated, *in vivo-*primed LN cells induced the expression of both arginase I and iNOS at the mRNA and protein levels (Figures 3A,B and Figure S1). The expression of both enzymes was the highest when 25% of iSN was used to stimulate infected MΦs, the same concentration that induced an optimal increase of PS exposure on intracellular amastigotes. However, iSN stimulation was weak, when compared to that in the positive controls for arginase I and iNOS expression, TGFβ_1_+IL-4 or IFNγ+TNFα, respectively (Figures 3A,B). The expression of both enzymes by activated MΦs correlates with the profile of cytokines present on those SNs, since they are constituted by a mix of Th1/Th2/modulatory cytokines (Figure 1D).

**Figure 4:**
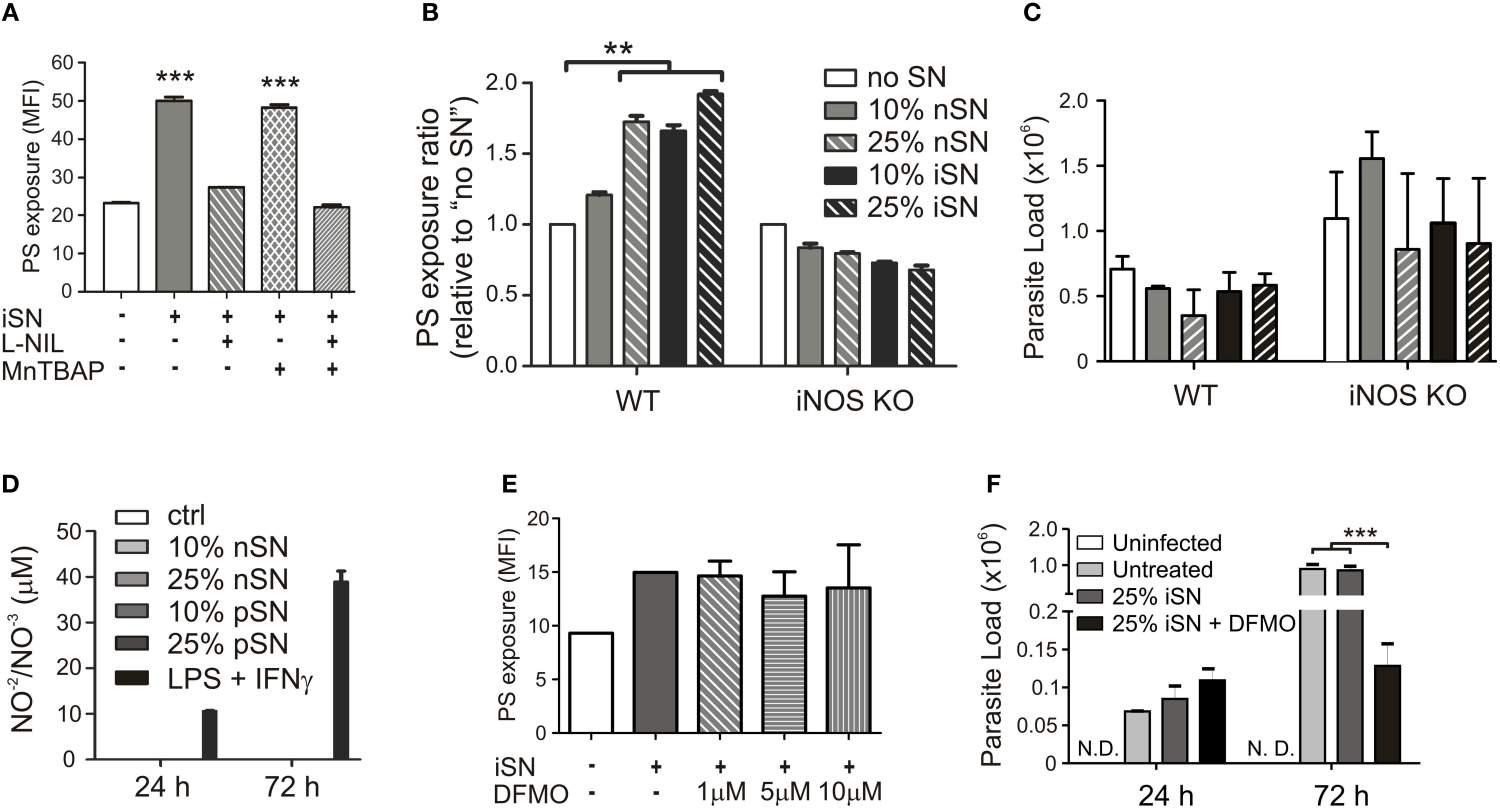
Cytokine-induced PS exposure on amastigotes depends on iNOS function. Infected BALB/c-derived BMMΦs were activated with SNs from re-stimulated, *in vivo*-primed LN cells (iSN) in the presence of **(A)** 50 μg/ml of L-NIL (a selective inhibitor of iNOS), 200 μM of MnTBAP (a superoxide scavenger molecule), or **(E)** different concentrations of DFMO (a selective ODC inhibitor). After 24 h of infection, amastigotes were purified for PS exposure analysis by flow cytometry. **(B)** BMMΦs were obtained from WT or iNOS KO mice, infected and activated with different concentrations of iSN and nSNs. After 24 h of infection, amastigotes were purified for PS exposure analysis by flow cytometry. Parasite loads in infected BMMΦs **(F)** treated with 10 μM of DFMO or **(C)** obtained from iNOS KO mice and activated with iSN were measured by real-time PCR. **(D)** NO production by BMMΦs stimulated with different concentrations of iSN, nSNs or LPS+IFN-γ (100 ng/ml for both), measured by Griess reaction. **(A)** Asterisks indicate comparison with untreated MΦs. Graphs represent data from 3 to 5 pooled experiments. ^**^p < 0.01, ^***^p < 0.001.

To understand the contribution of both pathways individually, we activated infected MΦs in the presence of L-NIL, a relatively selective inhibitor of iNOS, or, as a control, we used MnTBAP, a superoxide scavenger molecule. L-NIL has IC_50_ values of 0.4–3.3 μM for iNOS as opposed to 8–38 and 17–92 μM for eNOS and nNOS, respectively (Moore et al., 1994; Stenger et al., 1995). At 24 h of infection, we evaluated PS exposure on purified intracellular amastigotes. The iNOS inhibitor abrogated the cytokine-dependent induction of PS exposure, while scavenging superoxide molecules had no effect (Figure 4A). Treatment with both inhibitors brought the levels of PS exposure on amastigotes to the same levels of L-NIL alone, indicating that there was no synergistic effect between NO and superoxide to induce PS exposure on the parasite (Figure 4A). To further demonstrate the role of iNOS expression by MΦs in the induction of PS exposure on amastigotes, we generated BMMΦs from wild-type (WT) and iNOS^−/−^ C57BL/6 mice, infected them, and purified intracellular amastigotes from activated MΦs to evaluate PS exposure on the parasite. C57BL/6-derived MΦs were more sensitive to activation, since 25% of nSN was able to induce the same levels of PS on the parasite when compared with iSN (Figure 4B). Activation of infected iNOS^−/−^ MΦs did not increase PS exposure on amastigotes regardless of the source or the concentration of SN (Figure 4B). Similar to what happened in BALB/c-derived MΦs, the differences observed on amastigotes purified from WT or iNOS^−/−^ C57BL/6 MΦs were not related to the death of the amastigotes, since parasite loads 72 h post-infection were not altered among the different SN treatments (Figure 4C). Despite the increased iNOS expression, MΦ activation with 10 or 25% of iSN or nSN did not induce detectable amounts of NO, evaluated by Griess reaction (Figure 4D). To test whether polyamines play a role in the induction of PS exposure on amastigotes, we purified amastigotes from infected- and activated-MΦs treated with different concentrations of difluoromethylornithine (DFMO), an specific and irreversible inhibitor of ornithine decarboxylase, the enzyme responsible for metabolizing L-arginine-derived ornithine into the polyamine putrescine (Canellakis et al., 1979). Independent on the concentration of DFMO used, this drug had no effect on the PS exposure on the amastigotes (Figure 4E). However, when we quantified parasite loads on infected-MΦs activated with 25% of iSN, we observed that DFMO treatment was detrimental for the proliferation of intracellular parasites at 72 h post-infection (Figure 4F). This finding may indicate that polyamine synthesis is a requirement for parasite proliferation and maintenance in these conditions, as described in other models (Majumder and Kierszenbaum, 1993; Vannier-Santos et al., 2008). Nevertheless, our data imply that PS exposure on intracellular amastigotes is modulated by cytokine-mediated, iNOS-dependent MΦ activation, rather than by arginase I expression. Arginase I, however, is necessary for parasite proliferation and survival in activated MΦs.

### CD4^+^ T Cells Are Required for PS Exposure on Intracellular Amastigotes *in vivo*

**Figure 5:**
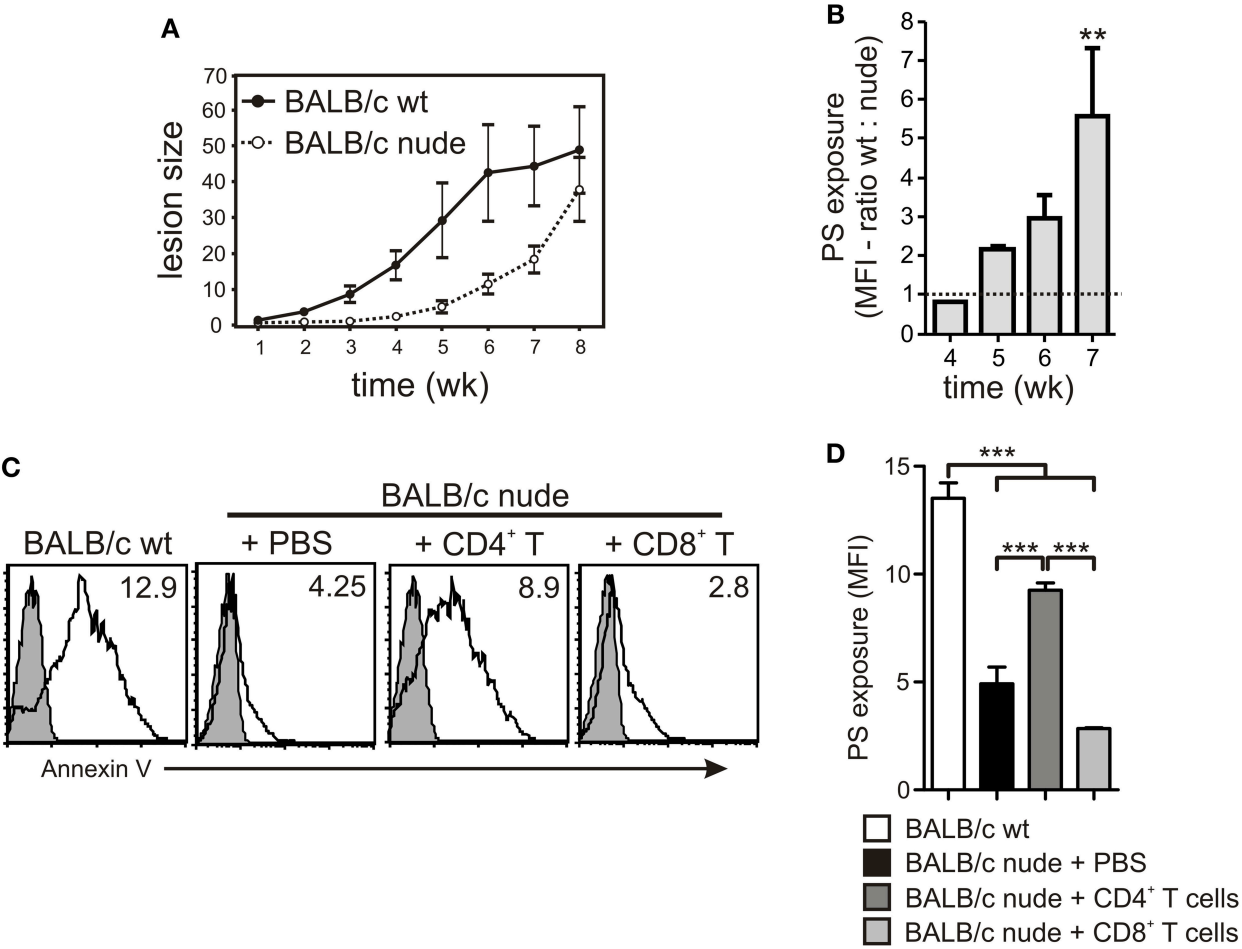
PS exposure on intracellular amastigotes is dependent on the presence of CD4^+^ T cells *in vivo*. **(A)** WT or nude BALB/c mice were infected with 2 × 10^6^ stationary-phase promastigotes on the hind footpad. Lesion sizes were measured weekly by using a Vernon caliper. **(B)** PS exposure on lesion-derived amastigotes was assessed by flow cytometry. Results are shown as the ratio between the MFIs of annexin V staining of amastigotes derived from WT vs. nude mice. **(C,D)** After 7–8 weeks of infection, 4 × 10^6^ CD4^+^ or CD8^+^ T cells, purified from draining LNs of infected WT BALB/c mice, were adoptively transferred to infected nude mice. At 2–3 weeks post-transfer, lesion-derived amastigotes were purified for PS exposure analysis by flow cytometry. **(C)** The shaded histograms represent amastigotes stained with annexin V in the absence of CaCl_2_. ^**^p < 0.01, ^***^p < 0.001. **(B,D)** Graphs represent data from 2 to 3 pooled experiments.

To further understand the role of different cellular populations present in the lymph nodes (LNs) for the modulation of PS exposure on intracellular amastigotes, we infected WT or nude BALB/c mice in the footpad and purified lesion-derived amastigotes to evaluate PS exposure on a weekly basis. Confirming previous data from the literature, we observed a delay in the development of lesions in immunodeficient mice, which, eventually, reached the same size of lesions from WT mice (Soong et al., 1997; Figure 5A). PS exposure on lesion-derived amastigotes purified from WT mice was 2- to 6-times higher than on parasites obtained from nude mice, depending on the time post infection (Figure 5B), confirming previous results (Franca-Costa et al., 2012). Since CD4^+^ T cells are the major regulators of host immune response to *Leishmania* infection, we hypothesized that the modulation of PS exposure on intracellular parasites observed by incubating infected MΦs with LN cells or culture SNs was due to CD4^+^ T cell-dependent MΦ activation. To test this hypothesis, we purified CD4^+^ T cells from draining LNs of infected WT mice and adoptively transferred these cells to infected nude mice. After 2–3 weeks post-transfer, we purified lesion-derived amastigotes to evaluate PS exposure. The adoptive transfer of CD4^+^ T cells increased PS exposure on the amastigotes by 2- or 3-fold, whereas transfer of CD8^+^ T cells, used as a control, did not alter the parasite phenotype (Figures 5C,D).

**Figure 6:**
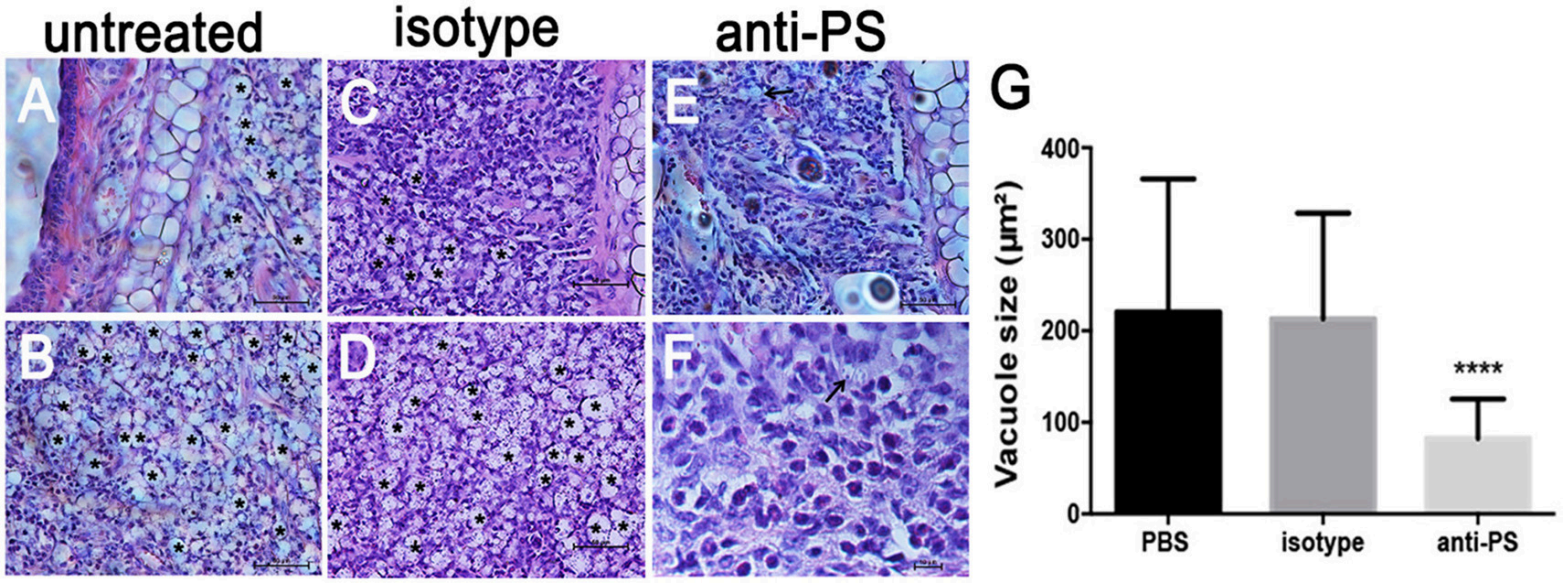
Histopathological analysis of mice lesions treated with anti-PS blocking monoclonal antibodies. **(A–F)** Mice were infected in the ear dermis with 10^6^ stationary-phase promastigotes. After 2 weeks of infection and every 3 days thereafter, mice were given i.p. injections of 100 μg of anti-PS or isotype control antibody. After 6 weeks of treatment the lesions were processed for histopathological analysis. **(A–D)** Asterisks in the images indicate large vacuoles containing amastigotes, **(E,F)** arrows indicate vacuoles containing parasites in anti PS treated infected mice. **(G)** Quantification of parasitophorous vacuole size from 2 independent experiments. ^****^p < 0.0001 compared to isotype and PBS treated mice.

In addition to the decreased PS exposure on amastigotes derived from nude mice (Franca-Costa et al., 2012; Figure 5B), histopathological analysis of BALB/c nude mice lesions showed a marked decrease in the size of the parasitophorous vacuoles of infected MΦs (Franca-Costa et al., 2012). This is also observed in lesions from MHC class II^−/−^ mice (Soong et al., 1997). We observed that infected mice treated intraperitoneally with anti-PS mAb displayed decreased parasitophorous vacuole size (Figures 6D–F) when compared to untreated mice (Figures 6A–C). The difference in the vacuole size was not observed in infected mice treated with isotype control antibodies (Figure 6G and Supplementary Table 1). Our data demonstrate that PS exposure on intracellular amastigotes is a response of the parasite when it senses MΦ activation through primed CD4^+^ T cells.

## Discussion

*L. amazonensis*, particularly its amastigotes, can infect host cells without triggering overt cellular activation. Actually, those parasites are able to down-modulate signaling pathways involved in dendritic cell activation, suppress cytokine production and expression of MHC class II molecules and, therefore, to inhibit antigen presentation (Prina et al., 2004; Xin et al., 2008). In addition, the inhibitory effects of *L. amazonensis* amastigotes on macrophages (MΦs) are also well known, including sequestration and degradation of MHC class II molecules, inhibition of endosomal proteases and blocking of NO production (Prina et al., 1990, 1993; Antoine et al., 1999). Consequently, *L. amazonensis* infection models are typically characterized by presenting a weak and non-polarized T cell response that is not sufficient to induce proper MΦ activation and control of parasite loads (Soong et al., 1997; Ji et al., 2002, 2003).

Our group has previously shown that the ability of amastigotes of *L. amazonensis* to silently infect host cells is mainly due to the exposure of PS molecules at their surface in a mechanism referred to as apoptotic mimicry (de Freitas Balanco et al., 2001; Wanderley et al., 2006). The recognition of this molecule and opsonizing ligands such as antibodies and complement factors mediate amastigote uptake by MΦs. In addition PS is highly effective in triggering anti-inflammatory cytokine production by MΦs, creating a permissive environment for parasite intracellular proliferation (de Freitas Balanco et al., 2001; Wanderley et al., 2006; Birge et al., 2016). Interestingly, PS exposure on amastigotes is correlated with disease severity, since amastigotes derived from BALB/c mice, which develop a more severe disease when exposed to higher amounts of PS than do those obtained from moderately susceptible C57BL/6 mice (Wanderley et al., 2006). In the context of parasite/host interactions, there are several reports showing a similar effect in the opposite direction: infection modulating host cell functions such as apoptosis, and expression of molecules involved with microbicidal effects or immune functions (Osorio y Fortea et al., 2007; Soong, 2008; Lecoeur et al., 2010; Muxel et al., 2017b). However, the presence of this phenotype suggests that, in the case of BALB/c mice infection, the host is able to modulate parasite virulence by inducing or selecting amastigotes with an increased capacity to expose PS. Definitely, this modulation depends on host cell types and activation status, since the internalization of axenically-cultured amastigotes by MΦs induces a basal level of PS exposure on these parasites, which is absent *in vitro*. However, MΦs with different genetic backgrounds are not able, by themselves, to differentially modulate PS exposure by the intracellular parasites. Differential modulation only occurs in the presence of lymph node (LN) cells or soluble molecules produced by antigen-stimulated LN cells.

PS recognition is clearly related to the internalization of amastigotes by MΦs (de Freitas Balanco et al., 2001; Wanderley et al., 2006) and to the modulation of dendritic cells and MΦ responses by both promastigotes and amastigotes (Wanderley et al., 2006, 2009, 2013; Franca-Costa et al., 2012). These events are dependent on PS recognition by surface receptors expressed by MΦs and other phagocytic cells. However, our results indicate that PS exposure on intracellular amastigotes is a counteraction of MΦ activation, suggesting that the parasite is able to modulate the host cells from within the parasitophorous vacuole. Therefore it should be determined whether there are endosomal PS receptors expressed by the MΦ, especially those ones involved in the regulation of host cell inflammatory activation such as TAM receptors (Axl, Tyro3, and Mer; Rothlin et al., 2007). Since signal transduction can be mediated by endosomal receptors it is likely to assume that PS receptors either coopted from the plasma membrane during parasite uptake or specifically trafficked to the parasitophorous vacuole.

It is known that the T cells response elicited by *L. amazonensis* infection is not sufficient to induce parasite killing, which was confirmed by our results. Cytokines produced by both *in vivo* primed (iSNs) or naïve LN cells (nSNs) stimulated with SLA were able to induce increased PS exposure, although this effect is prominent when *in vivo* primed cells were used, due to higher cytokine concentrations. In addition to this effect, we are demonstrating that MΦ stimulation with soluble factors produced by LN cells stimulated with *L. amazonensis* antigens generates a unique condition that triggers PS exposure on intracellular amastigotes, thereby increasing parasite virulence (Wanderley et al., 2006). We tried to activate infected MΦs with different combinations of cytokines, such as IFN-γ and TNF-α, in an attempt to create the exact conditions that drive PS exposure on the intracellular parasites. Although some induction of PS exposure was observed, it did not reach the same efficiency when compared to iSN (data not shown). We understand that PS exposure depends on a very unique combination of time-dependent and concentration-dependent cytokines, which favor parasite survival over parasite killing, but maintain stress-signals sufficient to induce PS exposure. This issue could be addressed by using SNs from LN cells from other infected mice strains, such as C57BL/6 or C3H.He mice (de Oliveira Cardoso et al., 2010). There are several reports demonstrating that CD4^+^ T cells are pathogenic for *L. amazonensis* infection; however, the mechanisms underlying this effect are not fully understood. It is well known that C57BL/6 mice deficient in the CIITA, MHC class II, and RAG2 genes or nude C57BL/6 mice exhibit a delay in lesion development and smaller parasite loads in infected tissues, indicating that CD4^+^ T cells play a role in lesion pathology and disease progression (Soong et al., 1997). However, those mice are persistently infected, developing lesions at later time points. The administration of competent CD4^+^CD25^+^ regulatory T cells is capable of transiently inhibiting those pathogenic effector cells, ameliorating the disease (Ji et al., 2005). Actually, amastigotes derived from BALB/c nude mice expose low amounts of PS, when compared to those in parasites obtained from their WT counterparts (Franca-Costa et al., 2012). The adoptive transfer of CD4^+^ T cells to infected nude mice stimulated PS exposure on lesion-derived amastigotes. These data reinforce the assertion that pathogenic CD4^+^ T cells affect *Leishmania* infection and suggest that these cells are necessary to generate amastigotes with high amounts of PS at their surface that, therefore, are highly capable of re-infecting new host cells, modulating MΦ functions and avoiding immune surveillance.

The outcome of *Leishmania* infection is determined by the efficiency of MΦ activation and by the enzyme metabolizing the aminoacid L-arginine. Classical MΦ activation through inflammatory cytokines leads to iNOS expression, NO production and parasite killing, whereas regulatory or anti-inflammatory cytokines lead to non-classical MΦ activation, arginase I expression, polyamine production and parasite survival and growth (Wanasen and Soong, 2008; Acuna et al., 2017; Muxel et al., 2017a). Both pathways use L-arginine as a primary substrate. Infected MΦs stimulated with supernatants from re-stimulated LN cells led to the simultaneous expression of iNOS and arginase I. This activation provides the necessary stimuli to increase PS exposure on intracellular amastigotes without interfering with the parasites' viability and proliferative capacity. The presence of very low concentrations of NO as a result of iNOS activation was sensed by intracellular amastigotes, triggering PS exposure, while arginase I expression and possible polyamine synthesis were necessary for maintenance of parasite viability and persistence in the host. NO is known to induce apoptosis in intracellular amastigotes (Murray and Nathan, 1999). However, it is possible that the low levels of NO produced, undetected by the Griess reaction, are sufficient to trigger PS exposure that does not lead to cell death due to the simultaneous presence of polyamines derived from arginase I/ODC activity. The latter can act as a protective factor, through DNA stabilization, protecting cells from DNA degradation or inducing autophagic processes, as shown in other models (Rowlatt and Smith, 1981; Ha et al., 1998; Madeo et al., 2010). However, our data do not exclude a possible participation polyamines derived from the parasite, since treatment with DFMO could block ODC expressed by the parasite. We are currently determining the optimal concentration of different NO-donor molecules, to induce PS exposure on axenic amastigotes. This information is necessary to study both the ability of NO to induce this phenotype and to better understand the role of polyamines for parasite survival. This mechanism configures a positive feedback cycle that is beneficial for the parasite. The poor activation of specific CD4^+^ T cell responses generates stimulatory conditions that induce non-classical MΦ activation, leading to concomitant and low expression of both iNOS and arginase I. Non-classical MΦ activation in turn, stimulates increased and sustained PS exposure on intracellular amastigotes, generating parasites more competent to infect new host cells and spread the anti-inflammatory signals derived from PS recognition. This mechanism seems to operate in BALB/c mice. The differential activation of CD4^+^ T cells in other mouse strains, such as C57BL/6, can explain the variations on PS exposure described in lesion-derived parasites from different mouse strains (Wanderley et al., 2006), and need to be further investigated. In addition, it is important to determine the impact of PS-dependent infection in human infections. Previous work showed that there is a positive correlation between PS exposure on parasites isolated from patients and the development of diffuse cutaneous Leishmaniasis (DCL). This correlation is also observed when comparing the level of PS on the surface of the isolated parasites and the number of lesions in the patient and the duration of the disease (Franca-Costa et al., 2012). DCL patients are characterized by low inflammatory and T cell response that leads to uncontrolled parasite dissemination and lesion development (Barroso et al., 2018). It is possible to suppose that in DCL patients, there is a unique combination of cytokines that induce augmented PS exposure on the parasite therefore leading to more severe disease.

One of the hallmarks of *L. amazonensis* infection is the peculiar parasitophorous vacuoles formed in infected MΦs. These vacuoles are large organelles shared by several parasites that continuously undergo fusion with lysosomes, exosomes and endosomes (Veras et al., 1996; Real et al., 2008). This feature is important for amastigotes to uptake nutrients (Borges et al., 1998), to dilute microbicidal molecules (Sacks and Sher, 2002; Wilson et al., 2008) and to evade the immune response (Antoine et al., 1999). Interestingly, different authors showed that enlarged vacuoles are less present in immunodeficient mice (Soong et al., 1997; Franca-Costa et al., 2012), suggesting that vacuole enlargement is also a counteractive response from amastigotes against a stressful environment. We observed that mice treated with anti-PS antibodies showed a marked and significant decrease in vacuole size when compared to untreated or isotype-treated mice. Surely, the antibodies are binding to released amastigotes since they do not have access to the parasitophorous vacuole. PS blockade could lead to a deviation in the endocytic pathway of parasite internalization since PS-dependent amastigote internalization occurs by macropinocytosis (Wanderley et al., 2006), which is characterized by the formation of enlarged endosomes (Basagiannis et al., 2016). The effect of anti-PS blocking antibodies on vacuole size provides a further explanation for the decreased parasite load of mice treated with anti-PS blocking antibodies (Wanderley et al., 2013). These results suggest that MΦ activation by T lymphocytes stimulate PS exposure and the consequences of this exposure are the alternative activation of MΦs, increase amastigote infectivity and enlargement of the parasitophorous vacuoles. The direct mechanism that link PS exposure and vacuole enlargement warrant further investigation.

In summary, this work describes that cytokine-dependent interactions between CD4^+^ T cells and infected MΦs are sensed by intracellular parasites, which counteract by exposing PS. Exposed PS, in turn, down-regulates the MΦ microbicidal capacity (Wanderley et al., 2006, 2013). Such cross-talk is obtained by a fine-tuned balance between iNOS activation, sufficient for stress-induced PS exposure, and arginase I activation, required for maintaining parasite survival and proliferation. We provide evidence that the increased PS exposure observed on amastigotes *in vitro* or from mouse lesions is due to MΦ stimulation by cytokines produced by CD4^+^ T cells. Therefore, the cellular immune response against the parasite can be exploited by the pathogen, generating amastigotes that are more competent to disseminate the disease and to escape from the host's immune system (Wanderley et al., 2006). In addition, we have provided further explanation for the pathogenic role of CD4^+^ T cells during *L. amazonensis* infection.

## Ethics Statement

This study was carried out in accordance with the recommendations of University of Texas Medical Branch Animal Care and Use Committee. The protocol was approved by the University of Texas Medical Branch Animal Care and Use Committee under the number #9803016A.

## Author Contributions

JW and PD performed *in vitro* infections, cell cultures, and *in vivo* adoptive transfer experiments. EC performed qPCR and western blot analysis of iNOS and arginase I expression. AP performed the anti-PS *in vivo* treatment and histopathological analysis. JW, RD, MB, and LS designed experiments, wrote the manuscript, and made helpful critiques.

### Conflict of Interest Statement

The authors declare that the research was conducted in the absence of any commercial or financial relationships that could be construed as a potential conflict of interest.
