# Supplementary material for: CD4+ T Cell-Dependent Macrophage Activation Modulates Sustained PS Exposure on Intracellular Amastigotes of Leishmania amazonensis
Source: Front Cell Infect Microbiol. 2019 Apr 12;9:105. doi: 10.3389/fcimb.2019.00105 (PMC6473175; doi:10.3389/fcimb.2019.00105)
Supplement: Supplementary file 1 [file Data_Sheet_1.PDF]

Supplementary table 1

	**PBS**	**Isotype control**	**anti-PS**
**Mean**	220,42	208,27	82,69
**SD**	145,36	114,36	43,32
N > 100 vacuoles per slide in each sample			
