# Supplementary figures and images for: CD4+ T Cell-Dependent Macrophage Activation Modulates Sustained PS Exposure on Intracellular Amastigotes of Leishmania amazonensis
Source: Front Cell Infect Microbiol. 2019 Apr 12;9:105. doi: 10.3389/fcimb.2019.00105 (PMC6473175; doi:10.3389/fcimb.2019.00105)

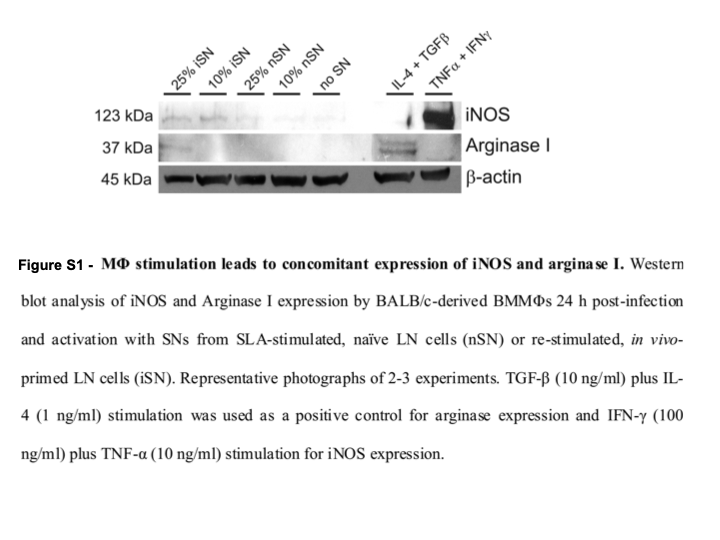

Supplement: Supplementary file 2 [file Image_1.TIF]
